# In‐Grain Ferroelectric Switching in Sub‐5 nm Thin Al_0.74_Sc_0.26_N Films at 1 V

**DOI:** 10.1002/advs.202302296

**Published:** 2023-06-29

**Authors:** Georg Schönweger, Niklas Wolff, Md Redwanul Islam, Maike Gremmel, Adrian Petraru, Lorenz Kienle, Hermann Kohlstedt, Simon Fichtner

**Affiliations:** ^1^ Department of Electrical and Information Engineering Kiel University Kaiserstrasse 2 D‐24143 Kiel Germany; ^2^ Fraunhofer Institute for Silicon Technology (ISIT) Fraunhoferstr. 1 D‐25524 Itzehoe Germany; ^3^ Department of Material Science Kiel University Kaiserstrasse 2 D‐24143 Kiel Germany; ^4^ Kiel Nano, Surface and Interface Science (KiNSIS) Kiel University Christian‐Albrechts‐Platz 4 D‐24118 Kiel Germany

**Keywords:** domains, ferroelectrics, neuromorphic computing, scandium, thin films

## Abstract

Analog switching in ferroelectric devices promises neuromorphic computing with the highest energy efficiency if limited device scalability can be overcome. To contribute to a solution, one reports on the ferroelectric switching characteristics of sub‐5 nm thin Al_0.74_Sc_0.26_N films grown on Pt/Ti/SiO_2_/Si and epitaxial Pt/GaN/sapphire templates by sputter‐deposition. In this context, the study focuses on the following major achievements compared to previously available wurtzite‐type ferroelectrics: 1) Record low switching voltages down to 1 V are achieved, which is in a range that can be supplied by standard on‐chip voltage sources. 2) Compared to the previously investigated deposition of ultrathin Al_1−x_Sc_x_N films on epitaxial templates, a significantly larger coercive field (*E*_*c*_) to breakdown field ratio is observed for Al_0.74_Sc_0.26_N films grown on silicon substrates, the technologically most relevant substrate‐type. 3) The formation of true ferroelectric domains in wurtzite‐type materials is for the first time demonstrated on the atomic scale by scanning transmission electron microscopy (STEM) investigations of a sub‐5 nm thin partially switched film. The direct observation of inversion domain boundaries (IDB) within single nm‐sized grains supports the theory of a gradual domain‐wall driven switching process in wurtzite‐type ferroelectrics. Ultimately, this should enable the analog switching necessary for mimicking neuromorphic concepts also in highly scaled devices.

## Graphical abstract

This study reports on sputter‐deposited ferroelectric sub‐5 nm thin Al_0.74_Sc_0.26_N. Scanning transmission electron microscopy (STEM) investigations reveal the formation of domain walls with a significant horizontal component and in‐grain partial switching on lateral dimensions below 10 nm^2^. The impact of the substrate type (silicon and sapphire) as well as a decrease in the coercive field (*E_c_*) with thickness are discussed in detail and enable switching voltages as low as 1 V.

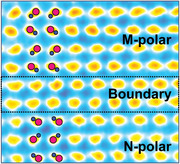

## 1 Introduction

In recent years, ferroelectrics have become one of the main foci of advancing semiconductor technology toward higher performance and energy efficiency.^[^ ^1^, ^2^, ^3^ ^]^ This applies especially to neuromorphic and in‐memory computing, where the field‐driven ferroelectric effect promises analog operation with the lowest input power. However, the entrance of ferroelectric functionality into the active areas of commercial devices other than binary ferroelectric random‐access memories (FRAMs) is yet to take place. One of the major challenges in this context is an excess of device‐to‐device variability of key parameters like the threshold voltage in small devices as well as the loss of their capability to operate in an analog fashion. This variability becomes pronounced when the ferroelectrically active area of a device approaches the size of the grains inside the ferroelectric films and the domains therein. This grain size typically is in the range of tens of nanometers for the fluorite‐type ferroelectrics, which have been at the focus of scientific attention in recent years.^[^ ^4^ ^]^ The factors contributing to device variability are a lack of crystalline texture, stress inhomogeneities, and less than complete phase purity, which lead to different material parameters between different grains.^[^ ^4^, ^5^ ^]^ For fluorite‐type ferroelectrics, the domain size can be considered equal to the grain size and thus has the same lower limit of tens of nanometers, which was shown to severely constrain analog operation in highly scaled devices.^[^ ^6^ ^]^

Since their discovery in 2019, the new wurtzite‐type ferroelectrics have raised expectations of a possible solution to the aforementioned issues.^[^ ^7^ ^]^ Wurtzite‐type ferroelectric films can typically be grown phase pure, well textured and the narrow distribution of their displacement current response upon ferroelectric switching promises a narrow distribution of the local ferroelectric properties, all of which should contribute to improved device repeatability. At the same time, wurtzite‐type films can easily be deposited at complementary metal oxide semiconductor (CMOS) back‐end‐of‐line (BEOL) compatible conditions, feature extreme temperature stability themselves and thicker films are already in large‐volume industrial production.^[^ ^8^, ^9^ ^]^ Further, for film thicknesses (*d*) above 100 nm, the switching kinetics of the material can be modeled to be domain‐wall motion‐limited,^[^ ^10^ ^]^ which suggests that more than a single state can be stored per grain. Despite these conceptual advantages, major challenges remain to be solved until the material class is able to fully meet the demands of advanced microelectronic devices. In this context, it is highly necessary to further reduce the ferroelectric switching voltage of wurtzite‐type thin films to meet the capabilities of typical on‐chip voltage supplies (in the range of 1 V), while retaining the aforementioned advantages like phase purity and domain‐wall motion‐limited switching.

In this study, we demonstrate for the first time that wurtzite‐type sub‐5 nm thin (8 to 9 unit cells corresponding to 4 – 4.5 nm) Al_0.74_Sc_0.26_N films sputter‐deposited on silicon (Si) substrates retain ferroelectric functionality with switching voltages as low as 1 V and feature in‐grain, nm‐sized domains upon partial switching. We were thus able to reduce the switching voltage and film thickness of films on Si by around 50% compared to literature (5 nm thin films grown by non‐BEOL compatible molecular beam epitaxy (MBE) and ≈10 nm thin films grown by sputtering on Si).^[^ ^11^, ^12^, ^13^ ^]^ Further, by performing atomic resolution scanning transmission electron microscopy (STEM) on epitaxial sub‐5 nm thin Al_0.74_Sc_0.26_N films, we obtained the first images of domain walls in any wurtzite‐type ferroelectric to confirm the presence of nm‐sized domains within individual grains.

Our investigation starts with a structural as well as an electrical comparison of 10 nm thin Al_0.74_Sc_0.26_N grown epitaxially on Pt/GaN/sapphire and grown non‐epitaxially on Pt/Ti/SiO_2_/Si to demonstrate the improved ferroelectric properties of the latter. Further downscaling to the sub‐5 nm range of ferroelectric Al_0.74_Sc_0.26_N films grown on Si is investigated. The scaling of the coercive voltage, including a decrease of *E*_*c*_ below 10 nm film thickness ultimately allowed to achieve switching voltages as low as 1 V and is discussed in detail. Epitaxial growth was investigated as well, as it allows to resolve the ferroelectric polarization reversal on atomic level in partially switched sub‐5 nm thin Al_0.74_Sc_0.26_N layers via STEM. The identification of regions with opposite polarity inside a single grain and the necessary occurrence of a domain boundary in between gives first insights into the size, shape, location, and evolution of ferroelectric domains in Al_0.74_Sc_0.26_N and potentially in the whole class of wurtzite‐type ferroelectrics.

## 2 Results and Discussion

### 2.1 Effect of Non‐Epitaxial Growth on Si versus Epitaxial Growth on GaN on the Ferroelectric Response of Al_0.74_Sc_0.26_N 

For the direct integration of ferroelectric wurtzite‐type films into CMOS technology, the possibility to deposit them on Si substrates without epitaxial templating is crucial. While one might assume that epitaxial growth and thus higher interface‐ and film quality will automatically result in improved electrical properties, this section motivates that the opposite can be the case for Al_1−x_Sc_x_N.

**Figure 1:**
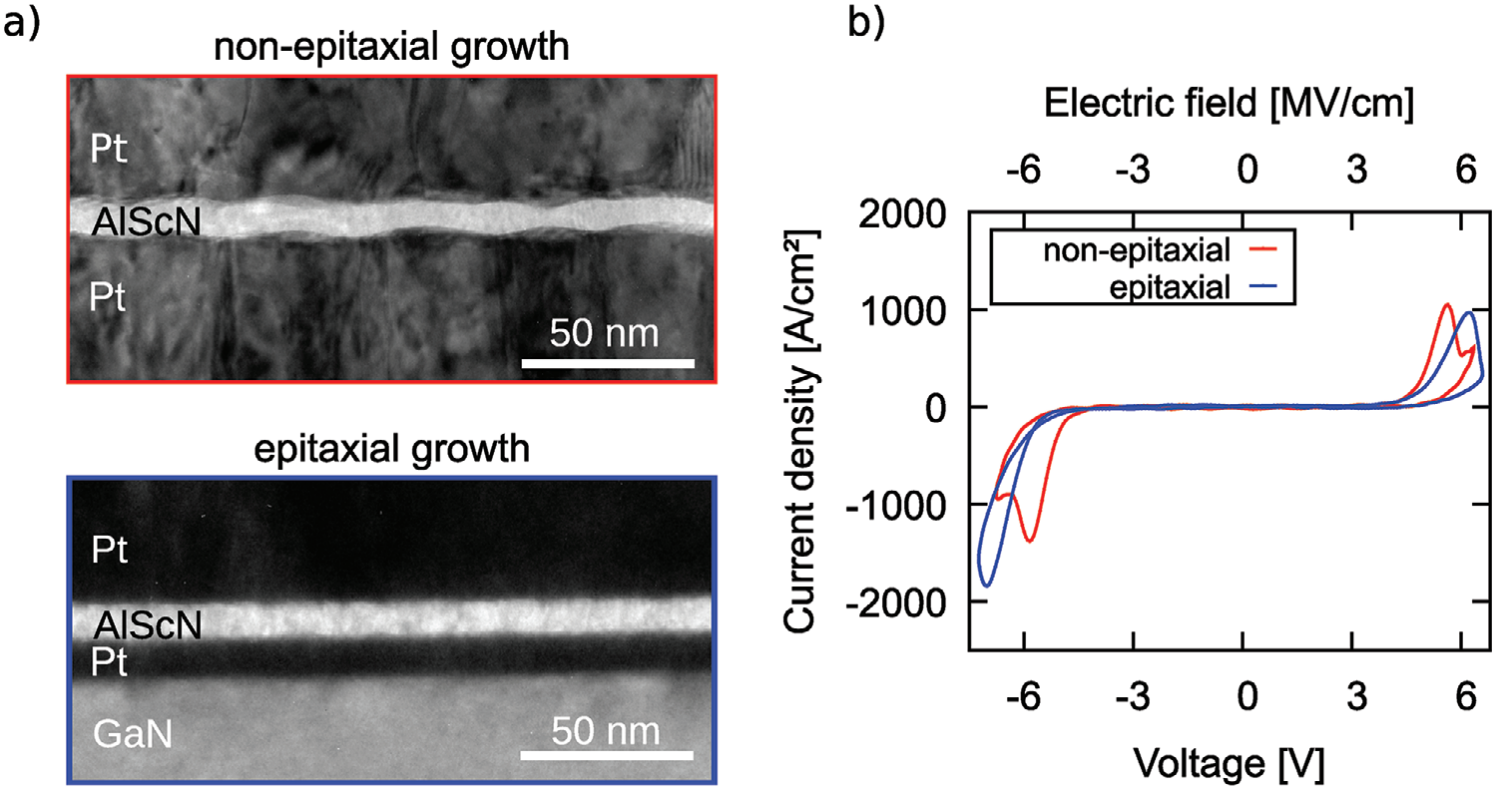
a) TEM cross‐section of 10 nm thin Al_0.74_Sc_0.26_N grown (top) non‐epitaxially within a Pt/Al_0.74_Sc_0.26_N/Pt/Ti/SiO_2_/Si and (bottom) epitaxially within a Pt/Al_0.74_Sc_0.26_N/Pt/GaN/sapphire capacitor stack. Only the capacitor structures are depicted. b) The *J* − *E* loops of the capacitors depicted in (a), measured at 100 kHz.

This can be concluded from the electrical response as well as from the interface quality investigations of 10 nm thin Al_0.74_Sc_0.26_N films. In **Figure** **1**a, the cross‐sections of 10 nm thin Al_0.74_Sc_0.26_N films grown epitaxially on Pt/GaN/sapphire as well as grown non‐epitaxially on Pt/Ti/SiO_2_/Si are compared. All films were capped in situ to prevent oxidation of the Al_0.74_Sc_0.26_N surface, which is crucial to obtain an undisturbed ferroelectric response especially of <10 nm thin films, where the thickness of the native oxide can be in the range of the total film thickness.^[^ ^14^, ^15^, ^16^ ^]^

For the epitaxially grown film stacks, the interfaces are smooth with an overall low surface roughness of the respective layers, which is known to result in a reduction of the leakage currents in capacitors.^[^ ^17^ ^]^ Structurally, the epitaxial films with a 10 nm thin Pt bottom electrode layer also have superior crystalline quality compared to non‐epitaxial ones, that is, higher *c*‐axis texture, which we investigate in detail in a separate work.^[^ ^18^ ^]^ Nonetheless, the films deposited on Si substrates exhibit more pronounced ferroelectric switching peaks (Figure 1b). Thus, despite their higher interface roughness and poorer interface texture compared to the epitaxial ones, a complete polarization inversion is demonstrated for the non‐epitaxial 10 nm thin Al_0.74_Sc_0.26_N grown on Pt/Ti/SiO_2_/Si, yet not for the epitaxial films. This is apparent from the drop in current density (*J*) after ferroelectric switching at the coercive field (*E*_*c*_) with maximum *J* followed by a local minimum before the contribution from leakage currents leads to a further increase in *J*. In comparison, no local minimum is observed for the epitaxial film. Although the leakage currents for the 10 nm thin epitaxial Al_0.74_Sc_0.26_N grown on Pt/GaN/sapphire are lower (at a fixed voltage) compared to films grown on Si, *E*_*c*_ is also higher and approaches the electrical breakdown field. Thus, as demonstrated in our recent work, we were able to fully switch the polarization of 10 nm thin epitaxial films via capacitance versus electric field (*C* − *E*) measurements, but not via *J* − *E* loops.^[^ ^14^ ^]^ We attribute the improved *E*_*c*_ of the films grown on Si compared to the ones grown on sapphire to differences in the respective Al_0.74_Sc_0.26_N film stress, which is well‐known to result in a shift of *E*_*c*_.^[^ ^7^ ^]^ Despite the fact that both heterostructures were grown under exactly the same Al_0.74_Sc_0.26_N deposition conditions (same run), the thermal expansion coefficients (*α*_*sub*_) of the silicon substrate (non‐epitaxial growth ‐ *α*_*sub*_ = 2.6 × 10^−6^ K^‐1^) and sapphire substrate (epitaxial growth ‐ *α*_*sub*_ = 7.3 × 10^−6^ K^‐1^) differ, leading to strong differences in the thermally induced film stress after cooling down from the Al_0.74_Sc_0.26_N (*α*_*film*_ = 4.9 × 10^−6^ K^‐1^) deposition temperature at 450 °C.^[^ ^19^, ^20^, ^21^ ^]^ The resulting strain (*ε*_*thermal*_) in the basal plane of Al_0.74_Sc_0.26_N can be calculated via Equation 1.

(1) $$\varepsilon_{thermal}=\int_{T_{RT}}^{T_{dep}}\left(\alpha_{film}-\alpha_{sub}\right)\;dT$$

For induced epitaxial strain an in‐plane lattice‐misfit of ≈4% between Al_1−x_Sc_x_N (*x* = 0.38) and GaN was calculated to result in a critical thickness of 2 nm.^[^ ^22^ ^]^ Therefore, we do not expect induced epitaxial strain at the Al_0.74_Sc_0.26_N/Pt interface (misfit ≈18%) for both types of substrates. Thus, in addition to the film stress induced by grain boundaries and defects, tensile stress is thermally induced in Al_1−x_Sc_x_N if grown on a silicon substrate ($\varepsilon_{thermal}\approx 0.09\%$), while compressive stress ($\varepsilon_{thermal}\approx -0.10\%$) is thermally induced if grown on a sapphire substrate.

The ability to tune the coercive field of Al_1−x_Sc_x_N exploiting the thermal expansion of varying substrates has also been reported recently by Yasuoka et al.^[^ ^23^ ^]^ In consequence, thermal induced tensile stress extends the in‐plane lattice resulting in the reduction of *E*_*c*_, similar to an increase in Sc concentration. To conclude, we attribute the more pronounced ferroelectric displacement current peak of the non‐epitaxial 10 nm thin Al_0.74_Sc_0.26_N to a more favorable position of *E*_*c*_ compared to the onset of leakage (compare the local minima in the current response) and with respect to the breakdown strength.

### 2.2 Ferroelectric Properties of Sub‐5 nm thin Al_0.74_Sc_0.26_N Films Grown on Si

Next, we present and discuss the electric characterization results of sputter‐deposited 8 to 9 unit cells (4 and 4.5 nm) thin Al_0.74_Sc_0.26_N films grown on Si wafers. Details on the exact thickness determination by using STEM can be found in Section 2.4.

**Figure 2:**
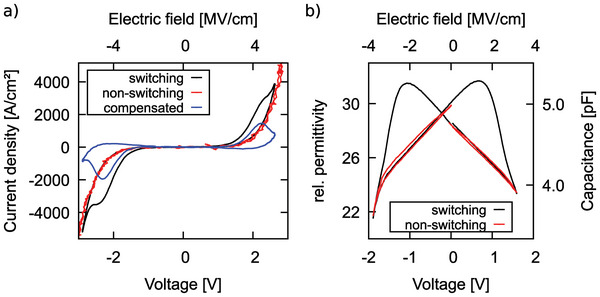
a) *J* − *E* loops of sub‐5 nm thin Al_0.74_Sc_0.26_N grown on Pt/Ti/SiO_2_/Si measured at 100 kHz on 5 µm diameter pads. A leakage current compensated curve (blue) by subtracting the non‐switching‐ (red) from the switching currents (black) is included. b) *C* − *V* loop of the sub‐5 nm thin Al_0.74_Sc_0.26_N based capacitor described in (a) measured on a 10 µm diameter pad. Unipolar non‐switching cycles (red) by measuring each branch (positive and negative voltages) twice with the same polarity are included to stress the non‐volatile nature of the permittivity enhancement due to ferroelectricity.

In **Figure** **2**a, *J* − *E* loops of sub‐5 nm thin Al_0.74_Sc_0.26_N grown on Pt/Ti/SiO_2_/Si are illustrated. In direct measurements (black curve), the clear hysteresis is already indicative of ferroelectric switching. The leakage current flow through the dielectric as well as the displacement current contributions due to the relative permittivity (*ε*_*r*_) can be separated from the hysteretic (i.e., ferroelectric) displacement currents by recording non‐switching loops (i.e., by pre‐poling the respective measured positive and negative branch). After subtraction of the non‐switching‐ (red curve) from the switching currents (black curve) the typical shape of ferroelectric displacement current peaks are obtained (blue curve), which allows the extraction of *E*_*c*_.

The *C* − *V* loop of the sub‐5 nm thin Al_0.74_Sc_0.26_N based capacitor depicted in Figure 2b further confirms the ferroelectric nature of the hysteretic event. A distinct butterfly‐shaped loop, typical for ferroelectric switching, is visible. Clearly distinguishable, non‐hysteretic non‐switching loops are depicted as well for the *C* − *V* loop. Furthermore, the polarization inversion of a sub‐5 nm thin film is unambiguously demonstrated by atomically resolved STEM investigations discussed in Section 2.4. Thus, it is demonstrated that such thin ferroelectric wurtzite‐type films can be grown by sputter deposition on oxidized silicon in a manner compatible with CMOS technology, which is a clear advantage over high‐temperature (⩾500 °C) MBE deposition processes on single crystal templates. ^[^ ^11^, ^15^ ^]^ Furthermore, the ferroelectric switching of Al_0.74_Sc_0.26_N films grown on silicon with, for wurtzite‐type materials, record low voltages down to 1 V is a major milestone toward ferroelectric Al_1−x_Sc_x_N based future devices operable with the on‐chip voltage supply of integrated circuits.^[^ ^24^, ^25^ ^]^

### 2.3 Coercive Field Scaling in Ultrathin Al_0.74_Sc_0.26_N 

The low switching voltage down to 1 V reached in our films is not only due to a simple reduction in thickness but also due to the favorable scaling of *E*_*c*_ with thickness, which we will therefore discuss in more detail in this section. In particular, the appearance of a depolarization field (*E_d_*) and its effects on the electrical response of films below 10 nm film thickness are discussed, as is the relative dielectric permittivity—which itself is related to the coercive field through the shape of the ionic potential wells.^[^ ^3^ ^]^

**Figure 3:**
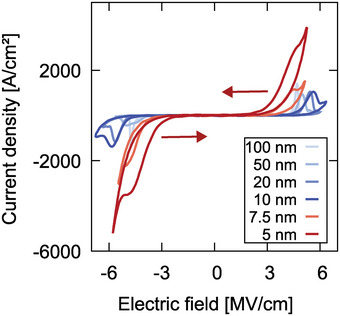
*J* − *E* loops of 100 nm‐ down to sub‐5 nm thin Al_0.74_Sc_0.26_N based capacitors deposited on Pt/Ti/SiO_2_/Si. All measurements were performed at 100 kHz on 5 µm diameter pads (< 10 nm Al_0.74_Sc_0.26_N thickness) and 10 × 10 µm^2^ pads (> 10 nm Al_0.74_Sc_0.26_N thickness). The decreasing trend of *E*_*c*_ with decreasing film thickness below 10 nm is indicated by red arrows.

In **Figure** **3**, *J* − *E* loops of 100 nm‐ down to sub‐5 nm thin Al_0.74_Sc_0.26_N based capacitors are depicted. From 100 nm down to 10 nm the coercive field is increasing with decreasing film thickness, but interestingly, below 10 nm the coercive field is significantly decreasing again, as indicated by the red arrows in Figure 3. A comparable trend with thickness scaling down to sub‐5 nm is also observed for epitaxial films grown on Pt/GaN/sapphire, although an almost thickness‐independent shift of Δ*E*_*c*_ ≈ 0.3 MV cm^‐1^ is visible (see Figure S2, Supporting Information). As discussed in the previous section, this shift in *E*_*c*_ results from the difference in thermally induced strain ($\Delta \varepsilon_{thermal}\approx 0.2\%$ for the different substrates), which fits well to the literature where similar *E*_*c*_ shifts for similar strain differences are reported.^[^ ^23^, ^26^ ^]^ Thus, it is concluded that the scaling properties are rather independent of the substrate (silicon vs sapphire), crystalline quality and associated growth modes (non‐epitaxial vs epitaxial).

**Figure 4:**
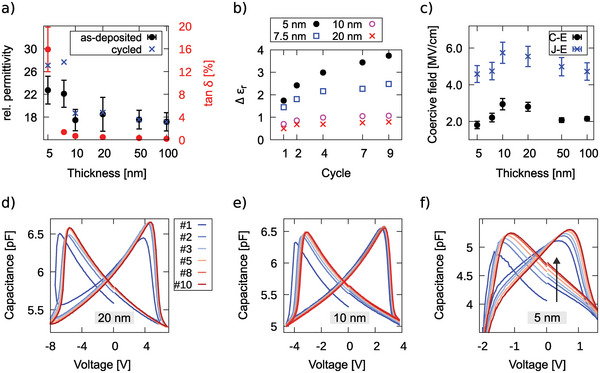
a) Relative permittivity as well as loss tangent as a function of Al_0.74_Sc_0.26_N film thickness for as‐deposited‐ and for pre‐cycled (10 times) capacitors grown on Pt/Ti/SiO_2_/Si. b) Absolute change of *ε*_*r*_ (state with full positive polarization, 0 V bias) with cycling in dependence of the Al_0.74_Sc_0.26_N film thickness. c) The coercive field dependence on Al_0.74_Sc_0.26_N thickness for the capacitors described in (a) determined via *J* − *E* (100 kHz) and via *C* − *E* (sweep time 20 s, small signal 100 mV and 900 kHz) loops. The coercive field determined via *C* − *E* loops is approximated by the peak positions of the butterfly‐loop. d) First ten *C* − *V* cycles of pristine capacitors consisting of 20 nm thin‐, e) 10 nm thin‐, and f) sub‐5 nm thin Al_0.74_Sc_0.26_N used for determining the change of *ε*_*r*_ with cycling as depicted in (b). The capacitor area was 695 µm^2^ (20 nm thickness), 341 µm^2^ (10 nm thickness), and 99 µm^2^ (sub‐5 nm thickness). The drop in capacitance at negative fields for the sub‐5 nm thin film depicted in (f) is due to the low and asymmetric resistivity (see Figure S4, Supporting Information).

A slight increase in *E*_*c*_ with decreasing film thickness down to 10 nm is consistent with the scaling properties reported so far for Al_1−x_Sc_x_N.^[^ ^11^, ^12^, ^13^, ^14^ ^]^ Yasuoka et. al. attributed this behavior to a change in the lattice‐parameters for thinner films due to stress gradients arising from the lattice mismatch between Pt (2.78 Å) and Al_1−x_Sc_x_N (3.22 Å for *x* = 0.2). Despite the high lattice mismatch, an epitaxial‐like growth between Pt grains of the bottom‐electrode layer and Al_1−x_Sc_x_N grains is suggested, eventually resulting in an increase in compressive strain in the basal plane when reducing the film thickness. In our films, the lattice‐parameters do not change significantly for thicknesses down to 10 nm. However, for sub‐5 nm thickness, the Al_0.74_Sc_0.26_N lattice‐parameters determined via STEM are *a* ≈ 319 pm and *c* ≈ 505 pm (details on the determination can be found in Experimental Section). This implies (relative to the equilibrium *a*‐lattice parameter of ≈324 pm at a Sc concentration of *x* = 0.26) an in‐plane compressive strain of ≈1.5%.^[^ ^26^, ^27^ ^]^ However, we measure *E*_*c*_ to decrease below 10 nm film thickness down to less than 2 MV cm^‐1^ in *C* − *E* curves, as illustrated in **Figure** **4**c. This decrease in *E*_*c*_ below 10 nm thickness differs from the very recently reported thickness scaling study down to 5 nm thin epitaxial films grown via MBE.^[^ ^11^ ^]^ In this work, Wang et al. also attributed the increase in *E*_*c*_ to a stress gradient forming due to the smaller in‐plane lattice‐parameter of the Mo bottom electrode compared to the one of Al_1−x_Sc_x_N.

The reduction in the electric field necessary for switching in films thinner than 10 nm is especially pronounced when considering the onset of the hysteresis opening in the *J* − *E* loops, as visible in Figure 3. For sub‐5 nm film thickness the hysteresis opens at 2.1 MV cm^‐1^, while for 100 nm film thickness, the opening starts at 4.3 MV cm^‐1^. This implies a more gradual switching capability below 10 nm film thickness.

A decrease in *E*_*c*_ in ultrathin ferroelectrics was reported by Dawber et al., who included depolarization field corrections into the Janovec–Kay–Dunn (JKD) scaling.^[^ ^28^, ^29^, ^30^ ^]^ The depolarization field resulting from a finite screening length in the electrodes adds up to the applied electric field if the condition 4π*P*_*s*_ > >*ε*_*r*_ *ε*_0_ *E* is fulfilled. For Al_0.74_Sc_0.26_N, with a spontaneous polarization (*P*_*s*_) of ≈110 µC cm^−2^ and electric fields up to 6 MV cm^‐1^, this condition is clearly satisfied (1507 >>14). Thus, similar to what was experimentally observed in ferroelectric PVDF films, a thickness dependent depolarization field qualitatively fits very well to the drop of *E*_*c*_ below 10 nm film thickness in Al_1−x_Sc_x_N.^[^ ^31^ ^]^ Very recently, it has also been demonstrated by first‐principles calculations that reducing the thickness of usually non‐switchable Wurtzite III–V semiconductors (e.g., AlSb) could result in polarization switching capability (i.e., ferroelectricity), due to the depolarization field which scales with ∝1/*d*.^[^ ^32^ ^]^

The decrease in *E*_*c*_ in our work is also reflected in the increase in *ε*_*r*_ below 10 nm film thickness, as illustrated in Figure 4a. In addition, *ε*_*r*_ increases to even higher values after cycling, which is especially pronounced for the thinner films (Figure 4b). A similar increase in the relative permittivity with cycling has also been observed for the wurtzite‐type ferroelectric Al_1−x_B_x_N.^[^ ^33^ ^]^ Through analysis of the Rayleigh parameters, this increase has been related to an increase in domain‐wall area compared to pristine samples at 0 V bias. If persistent domain walls indeed form during cycling and these domain walls extend vertically in the film, similar to what is reported in the following section, an enhancement in permittivity with lower film thickness would be a natural consequence ‐ due to an increase in the ratio of domain‐wall area to film volume with reduced thickness. This change in the ratio would imply a larger relative volume that is frustrated by the domain‐wall and in turn features a higher permittivity due to shallower ionic potential.

With decreasing film thickness not only the leakage current‐, but also the hysteretic area is increasing especially for sub‐5 nm film thickness, as illustrated in Figure 3. This increase in apparent displacement current can not be explained alone by polarization reversal, as it would imply a physically unlikely large spontaneous polarization above 1000 µC cm^−2^. The enhanced apparent polarization has therefore to be attributed to a dynamic current contribution triggered by the polarization reversal of the Al_0.74_Sc_0.26_N film. This is further supported by the fact that the remanent polarization determined via PUND measurements^[^ ^34^ ^]^ can be frequency depended until it reaches saturation at frequencies > 10^4^ Hz, as depicted in Figure S3 (Supporting Information). Currently, the most likely explanation of this behavior is the temporal formation of conductive domain walls during switching.^[^ ^35^ ^]^ For higher frequencies, the time domain walls are present in the sample is reduced, and therefore their contribution to the apparent switching polarization. As discussed above, with decreasing film thickness, the relative domain‐wall density will increase and domains are more likely to extend from the top to the bottom interface. Both effects can facilitate increased electrical current to flow in the form of compensation charges for the strong polarization discontinuity along the domain walls. This concept of conducting domains walls is closely related to the well‐known polarization doping schemes in III‐N semiconductors.^[^ ^36^, ^37^, ^38^ ^]^The model is further supported by non‐switching quasi‐static current response measurements demonstrating higher conductivity of partially switched capacitors at fields well below *E*_*c*_, and for both positive and negative fields (see Figure S5, Supporting Information). Further analysis of this effect is the focus of ongoing work.

### 2.4 Atomic Scale Investigation of Ferroelectric Domains in Sub‐5 nm thin Al_0.74_Sc_0.26_N

Analog‐like ferroelectric switching is an elegant approach for emulating synapses and thus a stable partially switched state is an essential material property in the context of neuromorphic computing.^[^ ^39^, ^40^ ^]^ Hence, it is important to image and understand the atomistic switching processes and the evolution of polarization discontinuities (i.e., ferroelectric domain walls). This section explores the microscopic consequences of ferroelectric switching in sub‐5 nm thin Al_0.74_Sc_0.26_N films as well as their general structural properties via high‐resolution STEM. The main focus of this study is the first observation of domain walls in individual Al_0.74_Sc_0.26_N grains in any wurtzite‐type ferroelectric. In order to clearly observe the local polarization on unit cell scale, the analysis was conducted on an epitaxial (in‐plane ordered), yet still polycrystalline film (0002 oriented columnar grains), which allows for direct imaging conditions because of the identical film/substrate crystallographic orientation. An overview image of the sub‐5 nm thin Al_0.74_Sc_0.26_N film showing individual epitaxial grains with *c*‐axis texture confirmed across the entire prepared area as well as *C* − *E* loops demonstrating the ferroelectric switching in 10 nm‐ down to sub‐5 nm thin epitaxially grown films is provided in Figures S1 and S6 (Supporting Information). While previous attempts to resolve the local polarization in wurtzite‐type ferroelectrics were successful to identify the polarization direction of single unit cells, the observation of domain walls has so far been elusive.

The epitaxial nature of the heterostructure allowed for an accurate thickness determination on the level of monolayers due to the atomically sharp interfaces (c.f., Figure 1a). Despite the columnar growth mode of sputtered films, the good structural quality of the in‐plane oriented growth enables the direct observation of the unit‐cell polarity within single grains on the atomic scale.^[^ ^41^ ^]^ In order to draw conclusions on the switching process itself (besides just confirming up and down polarization flips), the investigated capacitor was only partially switched from the nitrogen (N)‐polar to the metal (M)‐polar state. For this, the capacitor was pre‐switched to full N‐polarity by sweeping with a LCR‐meter (*C* − *V* measurement) to a positive field which is high enough to saturate the polarization reversal, with a subsequent sweep to a negative field which is ≈0.8 MV cm^‐1^ below the saturation point.

**Figure 5:**
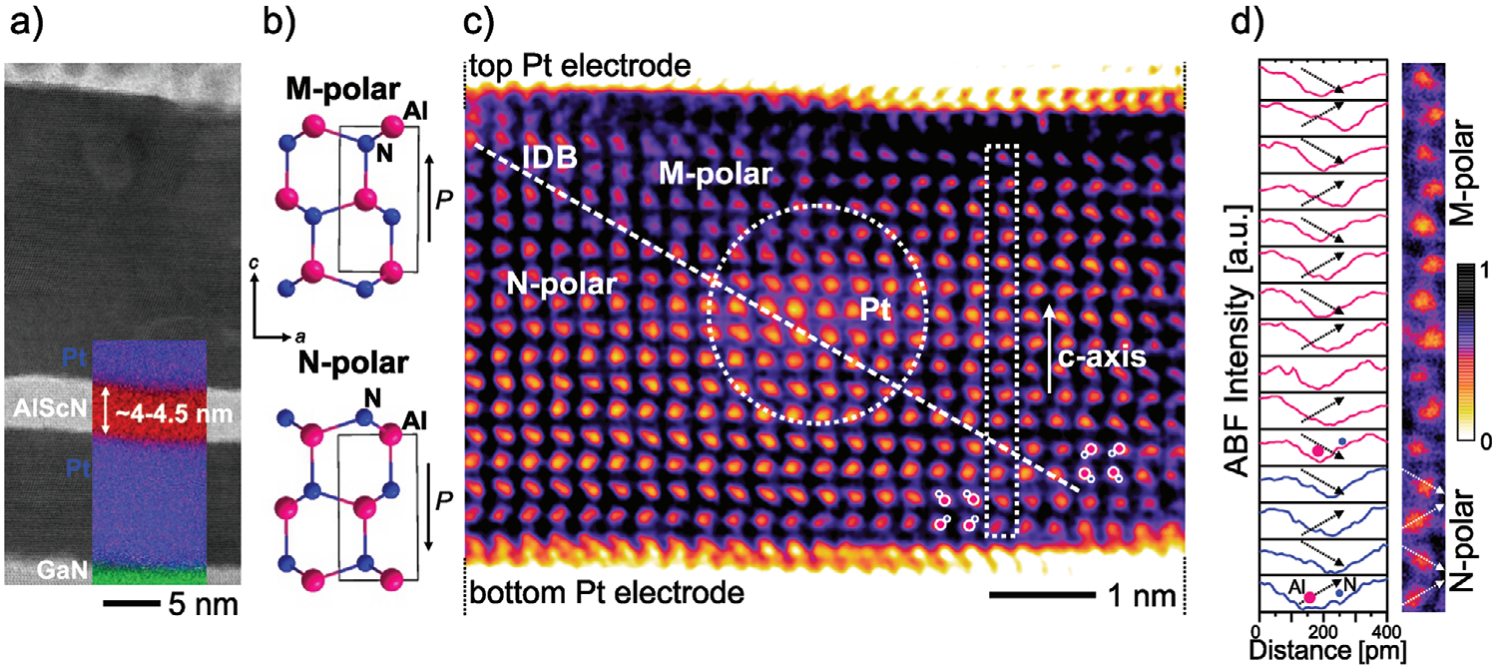
a) ABF‐STEM micrograph showing the Pt/Al_0.74_Sc_0.26_N/Pt/GaN capacitor stack in cross‐section. The inset shows the superimposed EDS maps of Pt, Al, and Ga. b) Sketches of the atomic structure in the M‐ and N‐polar state along the line of sight. c) Inverted‐ABF‐STEM micrograph of the full Al_0.74_Sc_0.26_N layer featuring an inclined inversion domain boundary separating regions of M‐polarity (upper right) and N‐polarity (lower left). Superimposed sketches of the (Al,Sc)‐N dumbbells help to visualize the polarization direction. d) Intensity profile analysis of the polarization direction of individual (Al,Sc)‐N dumbbells inside the single‐column frame. Profiles are always drawn from left to right (see arrows on the unfiltered single column image); color code: M(‐polarity) = pink, N(‐polarity) = blue.

The atomic scale STEM analysis of the partially switched capacitor is given in **Figure** **5**. An annular bright‐field (ABF)‐STEM micrograph of the capacitor cross‐section is depicted in Figure 5a. The individual thickness of the Pt electrodes is determined to be about 11 and 25 nm for the epitaxial Pt bottom electrode and the top electrode, respectively. They sandwich the Al_0.74_Sc_0.26_N layer with a total thickness of 8 to 9 unit cells, which was determined by counting the number of monolayers. This corresponds to 4 to 4.5 nm at a *c*‐lattice parameter of ≈505 pm. This number agrees well with the targeted thickness, considering the deposition rate calibrated on thicker films. Therefore, we conclude that there is no significant delay of film growth due to nucleation. The Sc content was verified by EDS analysis of a ≈10*x*4 nm^2^ frame to be *x* ≈ 0.26.

Atomic scale investigations of the polar domain structure were conducted using ABF‐STEM imaging paired with multi‐frame image alignment^[^ ^42^ ^]^ on the partially switched Al_0.74_Sc_0.26_N film. Here, the use of the ABF detector allows to routinely image atomic positions of light elements such as nitrogen, which is the crucial prerequisite to observe the polarity on the unit cell level in ferroelectric Al_1−x_Sc_x_N.^[^ ^41^ ^]^ Figure 5b depicts atomic models of the N‐ and M‐polar oriented wurtzite‐type structures sketched along the [2‐1‐10] viewing direction required for the investigation of unit cell polarity. As already discussed in related work,^[^ ^18^, ^26^, ^41^ ^]^ sputtered nanocrystalline films of Al_1−x_Sc_x_N exhibit small grain diameters of 2 – 6 nm featuring an in‐plane tilt between the individual grains in the order of 6° which restricts the observable area to single grains with exact orientation to the incident electron beam.^[^ ^14^ ^]^ In this respect, the ABF‐STEM image contrast formation crucially depends on exact orientation conditions.^[^ ^43^, ^44^ ^]^ In this investigation, the directly interpretable sample area was further limited by 1 to 2 nm large Pt agglomerates present evenly spaced in the center of the Al_0.74_Sc_0.26_N layer. These Pt artifacts were introduced during sample preparation using the FIB thinning method.

Individual grains with aligned zone axis orientation were identified in the Al_0.74_Sc_0.26_N layer after centering the GaN crystal lattice into the [2‐1‐10] orientation. The unit cell polarity was identified from the non‐rigidly registered multi‐frame ABF‐STEM data sets, by the analyses of intensity profiles drawn across the (Al,Sc)‐N dumbbells (see Figure S7, Supporting Information, for a demonstration on the GaN substrate). The ABF‐STEM micrograph contrast was inverted and a color scheme (inverted‐cABF‐STEM) was applied to enhance the image visibility as described in Experimental Section. No noise filter was applied for the analysis of intensity profiles to avoid potential artifacts by reducing the information limit. Intensity profile analysis is regularly performed to determine the polarity of materials with wurtzite‐type crystal structure because of the strong contrast difference between metal and nitrogen or oxygen atoms.^[^ ^45^ ^]^

**Figure 6:**
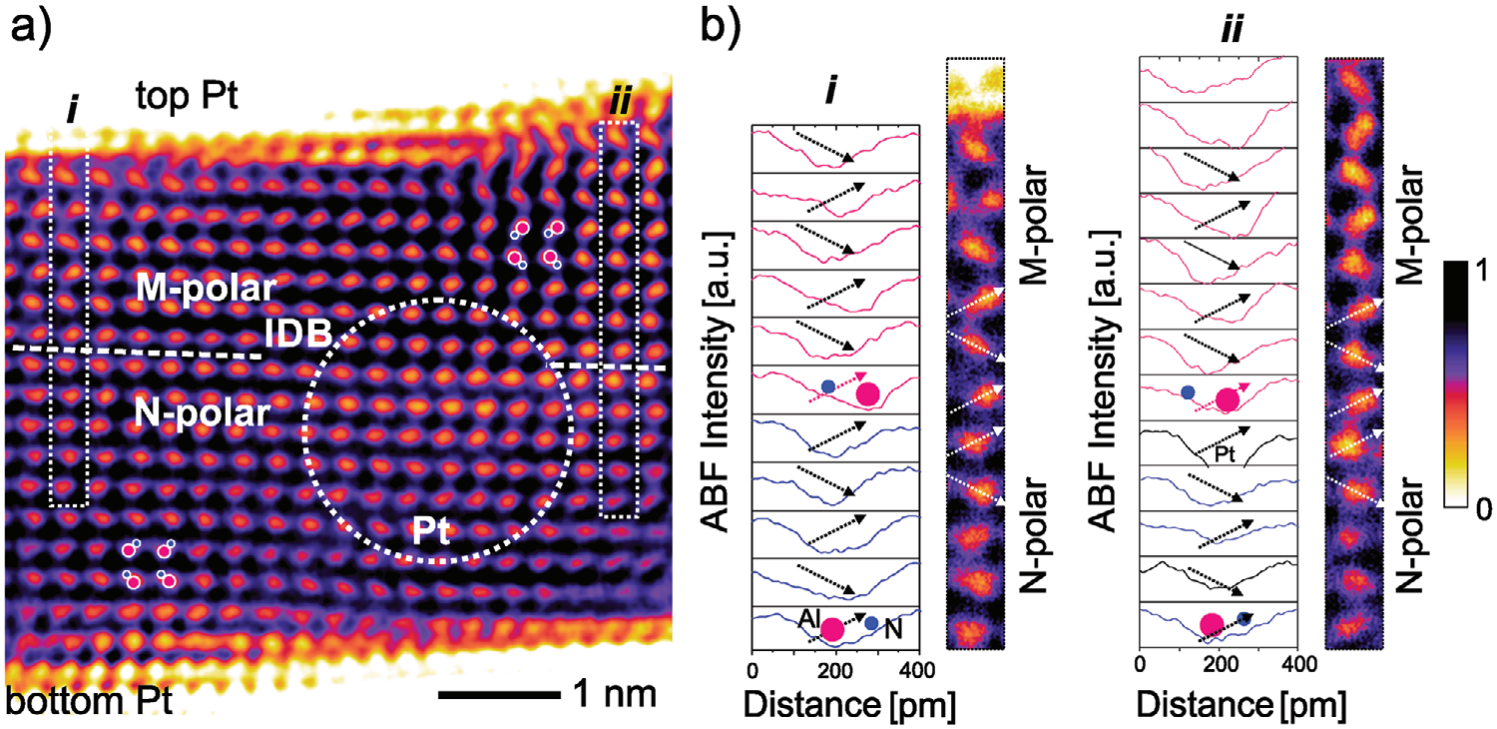
a) Inverted‐ABF‐STEM micrograph of the Al_0.74_Sc_0.26_N layer featuring a horizontal inversion domain boundary separating regions of M‐polarity within the top unit cells and N‐polarity in the bottom film. Sketches of the (Al,Sc)‐N dumbbells assist to visualize the polarity. b) Intensity profile analysis of the (Al,Sc)‐N dumbbells inside the vertical single‐column frames on the left (*i*) and right side (*ii*) of the grain. Both analyses hint toward the lateral progression of an IDB. Profiles are drawn from left to right (see arrows) on the unfiltered image; M(‐polarity) = pink, N(‐polarity) = blue.

Using the described method, the presence of N‐polar and M‐polar regions within a single grain is observed in Figures 5c and **6**. They confirm the presence of inversion domain boundaries (IDB) with a varying, yet always significant horizontal component. This is highly surprising given the fact that the horizontal component should give rise to an extreme polarization discontinuity at the domain‐wall, which likely requires an as‐yet‐unknown (charge) compensation or reconfiguration mechanism for stabilization. The nanocrystallinity of the film and thus the in‐depth superposition of slightly in‐plane rotated grains result in a less clear contrast compared to the single crystal material, for example, GaN. Furthermore, the expected 3D shape of the domains within the single grain itself adds certainly an additional level of diffusion to the atomic images and complicates the identification of the exact location of the IDB. On the other hand, the slightly in‐plane rotated grains ( 6°) permit to focus on individual grains and reduce the interference to neighboring grains in‐depth direction with a potentially different polarization state. Generally, M‐polarity is clearly identified in the upper region in the inverted‐ABF‐STEM images, while the remaining N‐polarity is predominantly located at the bottom interface. Figures 5d and 6b present the aforementioned profile analysis along the highlighted vertical atomic columns showing a clear N‐polar (blue profiles) polarization near the bottom interface and a switch to M‐polarity (pink profiles) closer to the upper interface. At the position of polarization inversion from N‐ to M‐polarity (the profiles are drawn in the scheme “up‐down” starting left of the (Al,Sc)‐N dumbbell), the alternating dumbbell orientation (and so the drawn profiles) is intercepted, hence the polarity abruptly inverses to the M‐polar state following an “up‐down‐down‐up” scheme as indicated by the arrows. This change of the polarization within the sub‐5 nm grains indicates that even in very thin films with grain diameters in the single‐digit nanometer range, Al_1–x_Sc_x_N can still feature more than two distinct polarization states. This suggests that the material, and possibly the wurtzite‐type ferroelectrics in general, are potentially very suitable for multi‐bit or even analog operation in single‐digit nanometer scaled devices. Further, as already assumed for thicker films,^[^ ^41^ ^]^ the nucleation of polar inversion domains switching from N to M‐polarity is found to be initiated at the top electrode interface and from there propagates toward the bottom interface. These results demonstrate the first direct observation of IDBs in wurtzite‐type ferroelectrics. From the application point of view, the in‐grain switching and small domain size is highly attractive to address multiple states in lateral dimensions < 10 nm^2^, which emphasizes the potential of Al_0.74_Sc_0.26_N for the realization of highly scaled synapse‐emulating neuromorphic computing devices.^[^ ^39^ ^]^

## 3 Conclusion

In summary, ferroelectric switching in sputter‐deposited, 8 to 9 unit cells (4 and 4.5 nm) thin Al_0.74_Sc_0.26_N grown non‐epitaxially on Pt/Ti/SiO_2_/Si and epitaxially on Pt/GaN/sapphire is demonstrated. The ferroelectric nature of the switching event was independently confirmed by electric *J* − *E* and *C* − *E* measurements, as well as by STEM investigations, resolving the polarization inversion at the atomic scale. This is the first report on a sub‐5 nm thin wurtzite‐type ferroelectric film switching fully on Si which also features record low switching voltages down to ≈1 V. Both aspects can be expected to greatly aid the future integration of the material class to advanced CMOS technology. Despite the better structural qualities of the thin film texture and interface structure of epitaxial films, the growth of sub‐5 nm non‐epitaxial Al_0.74_Sc_0.26_N on silicon results in an improved ratio between coercive‐ and breakdown field. Hence, the structural quality is not a limiting factor for good ferroelectric performance. *E*_*c*_ in our films increased only slightly with decreasing film thickness down to 10 nm, while it decreased when the film thickness is further reduced down to sub‐5 nm, thereby significantly lowering the required switching voltages. This behavior fits qualitatively to the depolarization‐corrected JKD model described by Dawber et al.,^[^ ^28^ ^]^ who explain the decrease in *E*_*c*_ by the increase in the depolarization field, resulting from the finite screening length of the electrodes. The increasing permittivity in thinner films supports this hypothesis. The permittivity is also found to increase with cycling, especially for thinner films, which we relate to an increase in the relative volume of domain walls with respect to the total film volume.

Our high resolution ABF‐STEM investigation of epitaxially grown Al_0.74_Sc_0.26_N for the first time allowed to resolve IDBs in a wurtzite‐type ferroelectric. The resulting presence of nanoscale domains spanning only fractions of individual nm‐sized grains suggests that domain‐wall motion still limits the switching kinetics in wurtzite‐type films thinner than 5 nm. The strong horizontal component of the observed domain walls further motivates the existence of a (charge) compensation mechanism for the strong polarization discontinuity at the boundary.

To conclude, the given evidence of in‐grain switching of sub‐5 nm thin films with sub‐5 nm lateral grain dimensions demonstrates stable partial switching capabilities of extremely low volumes. This switching mechanism together with the positive effects of thickness downscaling on *E*_*c*_ that result in ferroelectric switching voltages as low as 1 V make ferroelectric Al_1−x_Sc_x_N a highly interesting choice for nanoscale ferroelectric synaptic devices—as is the usage of a CMOS BEOL compatible deposition process already used in mass‐production.

## 4 Experimental Section

As electrodes, 100 nm thick Pt layers on a 10 nm thick Ti seed layer sputter‐deposited on SiO_2_/Si wafers were provided by Fraunhofer ISIT, Germany. Epitaxy‐ready templates consisting of GaN(4 µm)/sapphire were commercially bought. The substrates were diced into 1×1 cm^2^ chips with prior surface protection using a photoresist. Cleaning in acetone and isopropanol using an ultrasonic bath was performed, followed by rinsing in DI‐water. Subsequently, the non‐epitaxial Pt templates were cleaned by performing an Ar/O_2_‐plasma‐etching in a Sentech Sl100 reactor, details can be found elsewhere.^[^ ^46^ ^]^ The Al_0.74_Sc_0.26_N layers as well as the bottom epitaxial Pt and the Pt top layers were grown in‐house by sputter deposition using an Oerlikon (now Evatec) MSQ 200 multisource system, details about the process can be found in a previous publication.^[^ ^14^ ^]^ The epitaxial growth on Pt/GaN/sapphire as well as the non‐epitaxial growth on Pt/Ti/SiO_2_/Si was obtained by using the same deposition process. The Pt top layers were deposited in situ subsequently to the Al_0.74_Sc_0.26_N deposition after reaching a base pressure of at least 5 × 10^−7^ mbar. Pt was choosen as electrode material due to its high work function, resulting in a high Schottky‐barrier. This was expected to lower the leakage currents, which in turn facilitates the investigation of ferroelectric switching, especially in ultrathin films. Round‐ as well as square top electrodes were structured with lithography and ion‐beam etching (IBE, Oxford Instruments Ionfab 300). The dry‐etching was stopped right after the loss of Pt signal, detected via a secondary‐ion mass spectrometer (SIMS). The capacitance and loss tangent measurements were performed using a Hewlett Packard 4284 A Precision LCR meter. If not stated otherwise, the small signal voltage and frequency were 0.1 V and 900 kHz, respectively. The sweep time for *C* − *E* measurements of different Al_0.74_Sc_0.26_N thickness was kept constant by adjusting the delay time between each step, as well as the step width of the voltage sweep. The TEM investigated sample was pre‐switched by sweeping in 0.04 V steps with 100 ms delay in between. *J* − *E* measurements were performed using an AixACCT TF 3000 analyzer. A cross‐section sample of the partially switched film was extracted and thinned by the focused ion‐beam technique using a Helios600 FIB‐SEM machine and transferred into a JEOL (JEM200F) NEOARM scanning transmission electron microscope operated at 200 kV(cold‐FEG). Atomic scale investigation of the unit‐cell polarity within the sub‐5 nm Al_0.74_Sc_0.26_N layer was conducted using the ABF‐STEM mode with 10 to 20 mrad collection angle and a spatial resolution limit of ≈70 pm. To minimize the effects of scan distortions and sample drift during image acquisition, the atomic‐scale ABF‐STEM micrographs were recorded using fast serial recording of multi‐frame images followed by post‐processing image alignment using rigid and non‐rigid registration implemented in the Smart Align algorithm (HREM Research Inc.) on the DigitalMicrograph v.3.5.1 (DM) (GatanInc) software. If not stated otherwise, the non‐registered ABF‐STEM images were 1) Fourier filtered by a simple radiance difference filter using the lite version of DM plug‐in HREM‐Filters Pro/Lite v.4.2.1 (HREM Research Inc.) to remove high‐frequency noise, and 2) the ABF contrast was inverted, a color scheme was applied and the contrast was slightly enhanced within DM, for presentation purposes (inverted‐ABF‐STEM). The in‐plane and out‐of‐plane lattice parameters were estimated with ±2 pm accuracy by calculating the average atomic distance over minimum 8 and 6 unit cells, respectively, and are compared with the as‐determined lattice parameter of the GaN substrate. For GaN, the as‐determined lattice parameters are *a* ≈ 318 pm and *c* ≈ 521 and for Al_0.74_Sc_0.26_N these are *a* ≈ 319 pm and *c* ≈ 505. Chemical analysis on the capacitor stack was conducted using energy‐dispersive spectroscopy (EDS) with a dual silicon drift detector system with 100 mm^2^ active area each. The denoted scandium content *x* was defined as the number of Sc atoms relative to the total number of metal atoms (Sc + Al) with an estimated uncertainty of ±0.02. Cross‐section samples of 10 nm thin Al_0.74_Sc_0.26_N based capacitor structures grown on Pt/GaN/sapphire and grown on Pt/Ti/SiO_2_/Si were examined using a Tecnai F30 G^2^ STwin microcsope operated at 300 kV.

## Conflict of Interest

The authors declare no conflict of interest.

## Supporting information

Supporting Information

## Data Availability

The data that support the findings of this study are available from the corresponding author upon reasonable request.

## References

[1] A. I. Khan , A. Keshavarzi , S. Datta , Nat. Electron. 2020, 3, 588.

[2] T. Mikolajick , U. Schroeder , S. Slesazeck , IEEE Trans. Electron Devices 2020, 67, 1434.

[3] T. Mikolajick , S. Slesazeck , H. Mulaosmanovic , M. H. Park , S. Fichtner , P. D. Lomenzo , M. Hoffmann , U. Schroeder , J. Appl. Phys. 2021, 129, 100901.

[4] U. Schroeder , M. H. Park , T. Mikolajick , C. S. Hwang , Nat. Rev. Mater. 2022, 7, 653.

[5] M. Lederer , T. Kämpfe , R. Olivo , D. Lehninger , C. Mart , S. Kirbach , T. Ali , P. Polakowski , L. Roy , K. Seidel , Appl. Phys. Lett. 2019, 115, 222902.

[6] H. Mulaosmanovic , J. Ocker , S. Müller , U. Schroeder , J. Müller , P. Polakowski , S. Flachowsky , R. van Bentum , T. Mikolajick , S. Slesazeck , ACS Appl. Mater. Interfaces 2017, 9, 3792. 28071052 10.1021/acsami.6b13866

[7] S. Fichtner , N. Wolff , F. Lofink , L. Kienle , B. Wagner , J. Appl. Phys. 2019, 125, 114103.

[8] M. R. Islam , N. Wolff , M. Yassine , G. Schönweger , B. Christian , H. Kohlstedt , O. Ambacher , F. Lofink , L. Kienle , S. Fichtner , Appl. Phys. Lett. 2021, 118, 232905.

[9] R. Aigner , G. Fattinger , presented at *20th Int. Conf. on Solid‐State Sens., Actuators and Microsyst. Eurosensors*, Berlin, Germany 2019, pp. 523–526.

[10] S. Fichtner , F. Lofink , B. Wagner , G. Schönweger , T.‐N. Kreutzer , A. Petraru , H. Kohlstedt , presented at *2020 Joint Conf. of the IEEE Int. Frequency Control Sympos. and Int. Sympos. on Appl. of Ferroelectrics (IFCS‐ISAF)*, Keystone, CO, USA 2020, pp. 1–4.

[11] D. Wang , P. Wang , S. Mondal , M. Hu , D. Wang , Y. Wu , T. Ma , Z. Mi , Appl. Phys. Lett. 2023, 122, 052101.

[12] R. Mizutani , S. Yasuoka , T. Shiraishi , T. Shimizu , M. Uehara , H. Yamada , M. Akiyama , O. Sakata , H. Funakubo , Appl. Phys. Express 2021, 14, 105501.

[13] S. Yasuoka , R. Mizutani , R. Ota , T. Shiraishi , T. Shimizu , S. Yasui , Y. Ehara , K. Nishida , M. Uehara , H. Yamada , M. Akiyama , Y. Imai , O. Sakata , H. Funakubo , J. Ceram. Soc. Jpn. 2022, 130, 436.

[14] G. Schönweger , M. R. Islam , N. Wolff , A. Petraru , L. Kienle , H. Kohlstedt , S. Fichtner , Phys. Status Solidi‐RRL 2022, 17, 2200312.

[15] D. Wang , J. Zheng , P. Musavigharavi , W. Zhu , A. C. Foucher , S. E. Trolier‐McKinstry , E. A. Stach , R. H. Olsson , IEEE Electron Device Lett. 2020, 41, 1774.

[16] M. Li , K. Hu , H. Lin , V. Felmetsger , Y. Zhu , in 2022 IEEE Int. Ultrasonics Sympos. (IUS), IEEE, Piscataway, NJ, USA 2022.

[17] Y.‐P. Zhao , G.‐C. Wang , T.‐M. Lu , G. Palasantzas , J. T. M. D. Hosson , Phys. Rev. B 1999, 60, 9157.

[18] M. R. Islam , G. Schönweger , N. Wolff , A. Petraru , H. Kohlstedt , S. Fichtner , L. Kienle , under review. 10.1021/acsami.3c05305 37610983

[19] Y. Okada , Y. Tokumaru , J. Appl. Phys. 1984, 56, 314.

[20] W. M. Yim , R. J. Paff , J. Appl. Phys. 1974, 45, 1456.

[21] Y. Lu , M. Reusch , N. Kurz , A. Ding , T. Christoph , M. Prescher , L. Kirste , O. Ambacher , A. Žukauskaitė , APL Mater. 2018, 6, 076105.

[22] S. Zhang , W. Y. Fu , D. Holec , C. J. Humphreys , M. A. Moram , J. Appl. Phys. 2013, 114, 243516.

[23] S. Yasuoka , R. Mizutani , R. Ota , T. Shiraishi , T. Shimizu , M. Uehara , H. Yamada , M. Akiyama , H. Funakubo , ACS Appl. Electron. Mater. 2022.

[24] C. M. Maxfield , in FPGAs: Instant Access, Elsevier, Amsterdam 2008, pp. 13–48.

[25] X. Chen , N. A. Touba , in Electronic Design Automation, Elsevier, Amsterdam 2009, pp. 39–95.

[26] G. Schönweger , A. Petraru , M. R. Islam , N. Wolff , B. Haas , A. Hammud , C. Koch , L. Kienle , H. Kohlstedt , S. Fichtner , Adv. Funct. Mater. 2022, 32, 2109632.

[27] O. Ambacher , B. Christian , N. Feil , D. F. Urban , C. Elsésser , M. Prescher , L. Kirste , Jpn. J. Appl. Phys. 2021, 130, 045102.

[28] M. Dawber , P. Chandra , P. B. Littlewood , J. F. Scott , J. Phys.: Condens. Matter. 2003, 15, L393.

[29] H. F. Kay , J. W. Dunn , Philos. Mag. 1962, 7, 2027.

[30] V. Janovec , Czech. J. Phys. 1958, 8, 3.

[31] S. Ducharme , V. M. Fridkin , A. V. Bune , S. P. Palto , L. M. Blinov , N. N. Petukhova , S. G. Yudin , Phys. Rev. Lett. 2000, 84, 175. 11015863 10.1103/PhysRevLett.84.175

[32] C. Ke , Y. Hu , S. Liu , Nanoscale Horiz. 2023, 8, 616. 36945876 10.1039/d3nh00026e

[33] W. Zhu , F. He , J. Hayden , Z. Fan , J. I. Yang , J.‐P. Maria , S. Trolier‐McKinstry , Adv. Electron. Mater. 2021, 8, 2100931.

[34] S. Bernacki , L. Jack , Y. Kisler , S. Collins , S. D. Bernstein , R. Hallock , B. Armstrong , J. Shaw , J. Evans , B. Tuttle , B. Hammetter , S. Rogers , B. Nasby , J. Henderson , J. Benedetto , R. Moore , C. R. Pugh , A. Fennelly , Integr. Ferroelectr. 1993, 3, 97.

[35] D. M. Evans , V. Garcia , D. Meier , M. Bibes , Phys. Sci. Rev. 2020, 5, 20190067.

[36] D. Jena , S. Heikman , D. Green , D. Buttari , R. Coffie , H. Xing , S. Keller , S. DenBaars , J. S. Speck , U. K. Mishra , I. Smorchkova , Appl. Phys. Lett. 2002, 81, 4395.

[37] L. Yan , Y. Zhang , X. Han , G. Deng , P. Li , Y. Yu , L. Chen , X. Li , J. Song , Appl. Phys. Lett. 2018, 112, 182104.

[38] O. V. Khokhlev , K. A. Bulashevich , S. Y. Karpov , Phys. Status Solidi A 2013, 210, 1369.

[39] R. Islam , H. Li , P.‐Y. Chen , W. Wan , H.‐Y. Chen , B. Gao , H. Wu , S. Yu , K. Saraswat , H.‐S. P. Wong , J. Phys. D: Appl. Phys. 2019, 52, 113001.

[40] T. Shi , R. Wang , Z. Wu , Y. Sun , J. An , Q. Liu , Small Struct. 2021, 2, 2000109.

[41] N. Wolff , S. Fichtner , B. Haas , M. R. Islam , F. Niekiel , M. Kessel , O. Ambacher , C. Koch , B. Wagner , F. Lofink , L. Kienle , J. Appl. Phys. 2021, 129, 034103.

[42] L. Jones , H. Yang , T. J. Pennycook , M. S. J. Marshall , S. V. Aert , N. D. Browning , M. R. Castell , P. D. Nellist , Adv. Struct. Chem. Imaging 2015, 1, 1.

[43] D. Zhou , K. Müller‐Caspary , W. Sigle , F. F. Krause , A. Rosenauer , P. A. van Aken , Ultramicroscopy 2016, 160, 110. 26479323 10.1016/j.ultramic.2015.10.008

[44] E. Okunishi , H. Sawada , Y. Kondo , Micron 2012, 43, 538.

[45] M. de la Mata , C. Magen , J. Gazquez , M. I. B. Utama , M. Heiss , S. Lopatin , F. Furtmayr , C. J. Fernández‐Rojas , B. Peng , J. R. Morante , R. Rurali , M. Eickhoff , A. F. i Morral , Q. Xiong , J. Arbiol , Nano Lett. 2012, 12, 2579. 22493937 10.1021/nl300840q

[46] S. Fichtner , N. Wolff , G. Krishnamurthy , A. Petraru , S. Bohse , F. Lofink , S. Chemnitz , H. Kohlstedt , L. Kienle , B. Wagner , J. Appl. Phys. 2017, 122, 035301.

