# Supplementary material for: In‐Grain Ferroelectric Switching in Sub‐5 nm Thin Al0.74Sc0.26N Films at 1 V
Source: Adv Sci (Weinh). 2023 Jun 29;10(25):2302296. doi: 10.1002/advs.202302296 (PMC10477852; doi:10.1002/advs.202302296)
Supplement: Supplementary file 1 — Supporting Information [file ADVS-10-2302296-s001.pdf]

ADVANCED SCIENCE
Open Access

Supporting Information

for *Adv. Sci.*, DOI 10.1002/advs.202302296

In-Grain Ferroelectric Switching in Sub-5 nm Thin $Al_{0.74}Sc_{0.26}N$ Films at 1 V

Georg Schönweger, Niklas Wolff, Md Redwanul Islam, Maike Gremmel, Adrian Petraru, Lorenz Kienle, Hermann Kohlstedt and Simon Fichtner**

6 | SUPPLEMENT

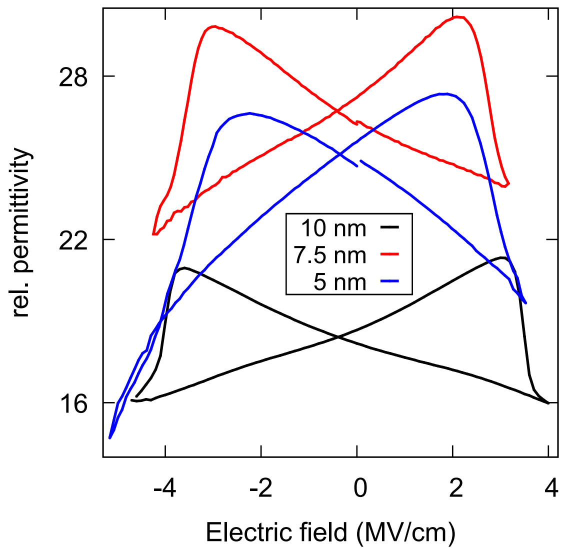

FIGURE S1 $C - E$ loops of 10 nm - down to sub-5 nm thin layers of $Al_{0.74}Sc_{0.26}N$ deposited epitaxially on Pt/GaN/sapphire. The sweep time for each thickness was kept constant. All measurements were performed on 10 µm diameter pads. No clear trend or ε_r with thickness can be observed from 10 nm down to sub-5 nm film thickness due to the error in relative permittivity of $\Delta(\varepsilon_r) \approx 4$, which mainly arises from variations in the capacitor area (nominal 10 µm diameter pads).

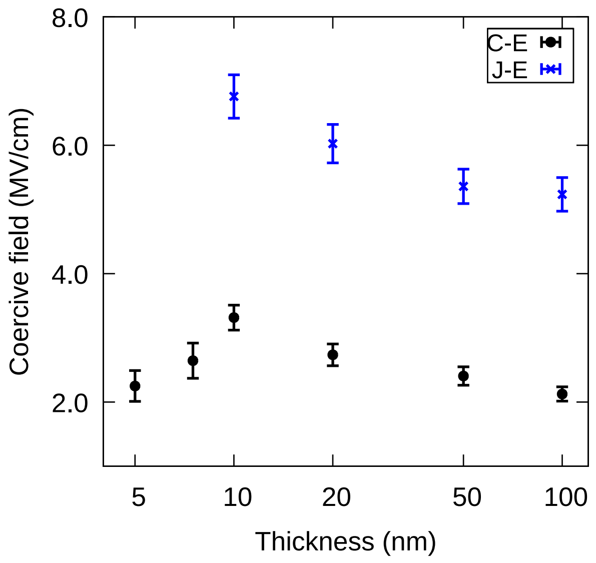

FIGURE S2 The coercive field of $Al_{0.74}Sc_{0.26}N$ epitaxially grown on Pt/GaN/sapphire in dependence on film thickness. E_c is determined via $J - E$ (80 kHz) and via $C - E$ (sweep time 20 s, small signal 100 mV and 900 kHz) loops. The breakdown field approaches the coercive field below 10 nm film thickness at high frequencies, thus not allowing to clearly determine E_c below that film thickness.

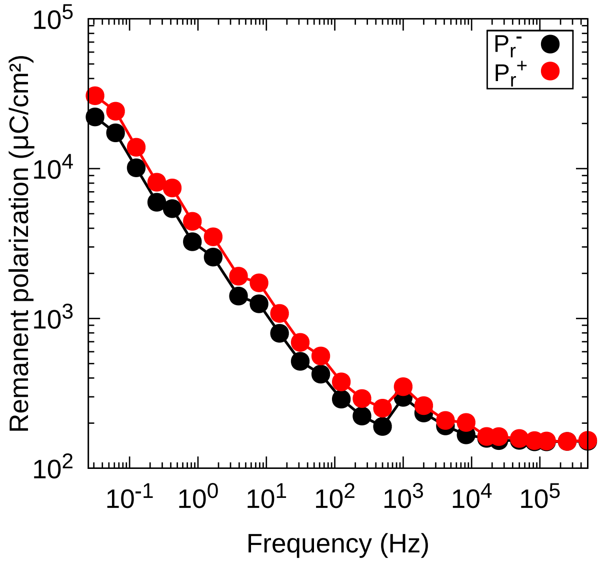

FIGURE S3 The remanent polarization of a 100 nm thin $Al_{0.74}Sc_{0.26}N$ based capacitor grown on $Pt/Ti/SiO_2/Si$ in dependence of the measurement frequency. The positive-up negative-down (PUND) method[34] was used to correct for non-hysteretic leakage- as well as capacitive currents. The P_r values for positive half-loops (P_r^+) and negative half-loops (P_r^-) are determined separately. At higher frequencies, the displacement current due to polarization reversal increases, while leakage current flowing through conduction paths such as through domain walls should be time independent. Therefore, the apparent remanent polarization, which amounts to the integration over time of both currents, saturates at higher frequencies.

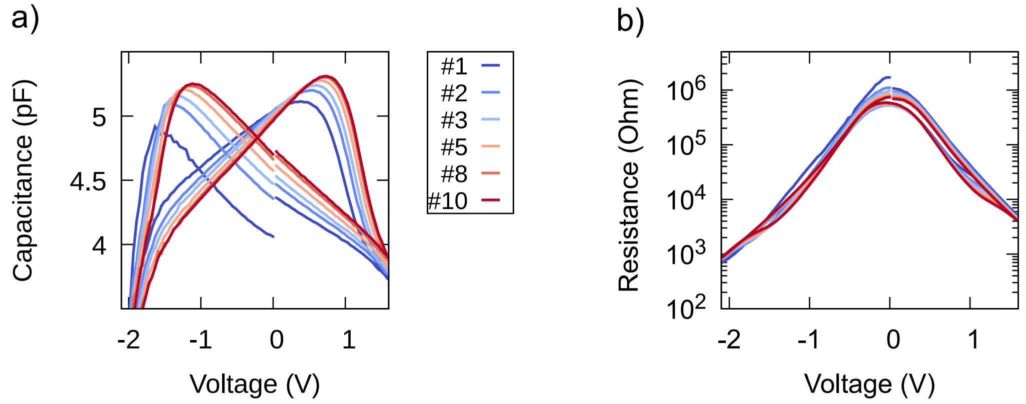

FIGURE S4 a) First ten $C-V$ cycles of pristine sub-5 nm thin $Al_{0.74}Sc_{0.26}N$ based capacitors grown on $Pt/Ti/SiO_2/Si$. b) Resistance of the respective curves shown in a). The decrease of the resistance to values below 1 kOhm at higher negative fields results in a capacitance drop.

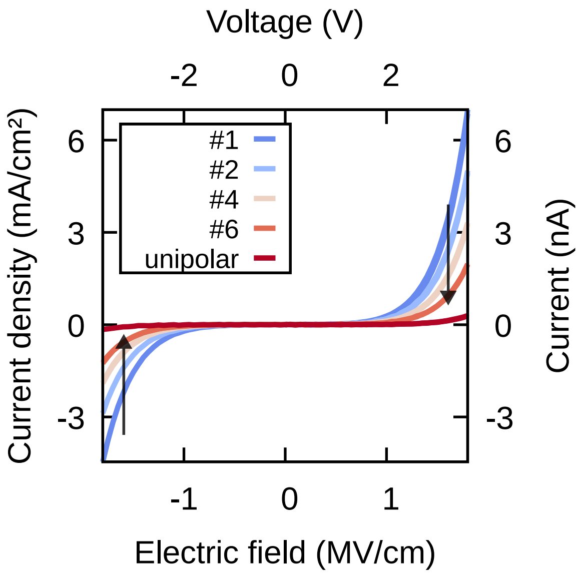

FIGURE S5 Quasi-static current response of minor non-switching loops (max. applied electric field below $E_c/2$) of a 20 nm thin $Al_{0.74}Sc_{0.26}N$ based capacitor grown on Pt/GaN/sapphire in dependence of the switching state. In the partially switched state, the capacitor is in a low resistance mode (blue curve). When switching more and more volume towards an unipolar state (transition from blue- to red curve, as indicated by the arrows), the resistance increases. This increase in resistance goes hand in hand with a decrease of ε_r. Both effects fit well to the model of conductive domain walls forming during switching - the domain-wall density decreases when switching the film partially towards the unipolar state and so does the current which flows through the conductive domain walls. The voltage was swept in steps of 0.1 V with a delay time between each step of 400 ms.

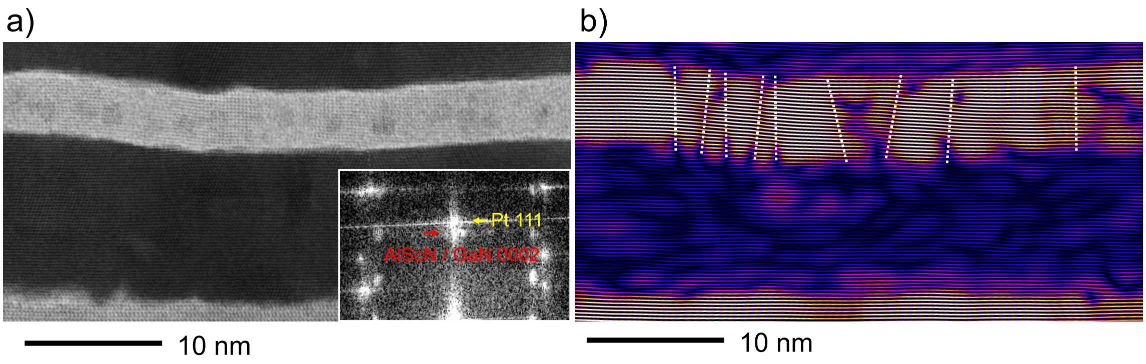

FIGURE S6 Nanoscale texture analysis of single-digit nanometer large columnar grains. a) ABF-STEM micrograph of the film cross section with Fast Fourier Transform (FFT) showing the aligned out-of-plane Pt 111 and $Al_{0.74}Sc_{0.26}N$ 0002 reflections. b) Inverse FFT image using an aperture to filter the spatial frequencies of the GaN and $Al_{0.74}Sc_{0.26}N$ out-of-plane 0002 reflections to demonstrate the crystal size and c-axis alignment. Dotted lines indicate the position of grain boundaries.

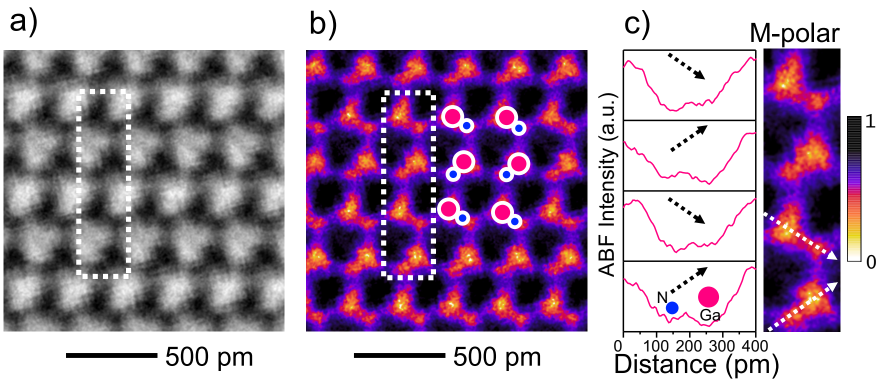

FIGURE S7 a) Unfiltered ABF-STEM micrograph of the GaN substrate and b) Inverted-ABF-STEM image featuring M-polarity. Sketches of the Ga-N dumbbells assist to visualize the M-polarity. c) Display of intensity profile analysis of the Ga-N dumbbells inside the vertical frame. Profiles are drawn from left to right (see arrows) on the unfiltered image; M(-polarity) = pink, N(-polarity) = blue.

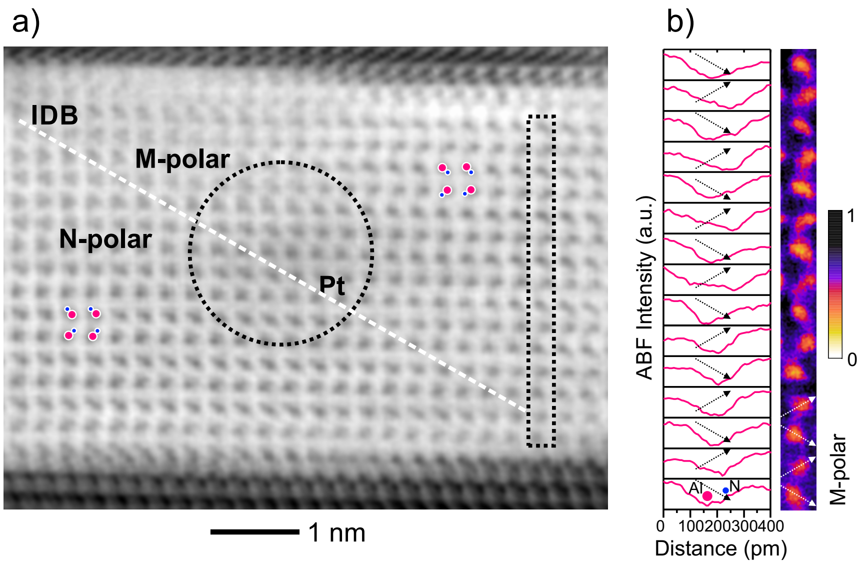

FIGURE S8 a) Unfiltered grayscale ABF-STEM micrograph of the inverted-ABF-STEM image shown in Figure 5 including the sketched position of the inclined inversion domain boundary. b) Profile analysis from the vertical single column frame where the M-polar domain reaches down to the bottom Pt interface. Profiles are drawn from left to right (see arrows) on the unfiltered image; M(-polarity) = pink, N(-polarity) = blue.
